# Complete Chromosomal Genome Sequence of Mycobacterium sp. Strain IWGMT90018-18076

**DOI:** 10.1128/mra.00078-22

**Published:** 2022-05-09

**Authors:** Hanako Fukano, Mitsunori Yoshida, Yuko Kazumi, Nobuya Sakagami, Manabu Ato, Satoshi Mitarai, Yoshihiko Hoshino

**Affiliations:** a Department of Mycobacteriology, Leprosy Research Center, National Institute of Infectious Diseases, Tokyo, Japan; b SRL, Inc., Tokyo, Japan; c Department of Mycobacterium Reference and Research, Research Institute of Tuberculosis, Japan Anti-Tuberculosis Association, Tokyo, Japan; University of Maryland School of Medicine

## Abstract

We have isolated a strain that we believe is identical to strain IWGMT90018—an unidentified nontuberculous mycobacterium (NTM) species published in 1981—and named it IWGMT90018-18076. Here, we report its complete chromosomal genome sequence. This study will help us understand the diversity and pathogenicity of NTM.

## ANNOUNCEMENT

Mycobacterium sp. strain IWGMT90018 is a slow-growing nontuberculous mycobacterium (NTM). This strain was first reported in 1981 in samples collected from a lung disease patient in Japan (1). The patient’s lungs had multiple cavities and large bullae in the lobes (2). The 16S rRNA sequence showed that the strain was similar to the Mycobacterium gordonae complex (3).

We isolated the unidentified NTM strain from a patient in Japan in 2018. The 16S rRNA gene sequence data and the results of biochemical tests were completely identical to those of IWGMT90018, isolated in 1981. So, we named the strain isolated in 2018 IWGMT90018-18076 and performed whole-genome sequencing on it (=JCM 34837, =KCTC 49725).

The strain was subcultured in Middlebrook 7H9 medium supplemented with 10% oleic acid-albumin-dextrose-catalase (OADC) enrichment at 37°C for 14 days. Genomic DNA for short-read Illumina sequencing was extracted using a NucleoSpin plant II kit (Macherey-Nagel, Germany), and that for long-read sequencing was extracted using a standard phenol-chloroform extraction technique. A paired-end sequencing library was prepared using a Nextera XT DNA sample prep kit (Illumina, Inc., San Diego, CA) according to the manufacturer’s instructions. Illumina paired-end (2 × 150-bp) reads (1,275,958) were obtained using the HiSeq X system (Illumina) and assessed using FastQC ver. 0.73 (4).

Long sequence reads were obtained using a MinION device (Oxford Nanopore Technologies, Oxford, UK). Short DNA fragments were removed using the short-read eliminator kit (Circulomics, Baltimore, MD). We prepared the sequencing library using the rapid barcoding sequencing library kit (SQK-RAD004) following the manufacturer’s protocol and performed sequencing on an R9.4.1 flow cell. The raw sequence data (FAST5 format) were base called using Guppy ver. 4.0.11. The quality of the Nanopore reads was assessed using NanoPlot ver. 1.28.2, and a total of 163,347 reads (total number of bases, 962,879,294; read length *N*_50_, 14,558 bp; mean read quality, 10.1) were generated.

The Nanopore sequence reads were *de novo* assembled into a contig using Flye ver. 2.8.3 with the “–nano-raw” setting (https://github.com/fenderglass/Flye). The assembled contig was circularized manually using GENETX-MAC ver. 21.0.0. The assessed short-read sequences were mapped to the circularized chromosomal contig using minimap2 ver. 2.20 and polished using Pilon ver. 1.20.1 (5, 6). The resulting sequence was rotated and annotated using DFAST ver. 1.1.15 (7). Default parameters were used for all software unless otherwise specified.

The chromosome of strain IWGMT90018-18076 comprises 6,346,450 bp (G+C content, 67.1%; coverage, 96×). The number of predicted coding sequences (CDSs), rRNA operons, and tRNAs in the genome was 6,263, 3, and 55, respectively.

The average nucleotide identity (ANI) values of the M. gordonae complex were calculated using FastANI ver. 1.3.

The ANI values between the genomes of strain IWGMT90018-18076 and those of M. gordonae DSM 44160^T^ (GenBank accession number LQOY01000000), Mycobacterium paragordonae strain 49061^T^ (NZ_CP025546.1), Mycobacterium vicinigordonae strain 24^T^ (NZ_CP059165.1), and Mycobacterium asiaticum (NZ_MVHI00000000.1) were 82.16%, 82.46%, 82.16%, and 81.92%, respectively. These results indicated that strain IWGMT90018-18076 was likely an independent species of the M. gordonae clade.

The complete genome sequence of strain IWGMT90018-18076 comprises essential data for understanding the diversity and pathogenicity of NTM.

### Data availability.

The data have been deposited in the DDBJ/EMBL/GenBank databases under accession number AP025179.1 for the chromosome. The Illumina paired-end and ONT base-called fastq files are available in the Sequence Read Archive under accession numbers DRR332975 and DRR332976, respectively.
